# A unified approach for cluster-wise and general noise rejection approaches for k-means clustering

**DOI:** 10.7717/peerj-cs.238

**Published:** 2019-11-18

**Authors:** Seiki Ubukata

**Affiliations:** Graduate School of Engineering, Osaka Prefecture University, Sakai, Osaka, Japan

**Keywords:** Clustering, k-means, Noise rejection, Rough set theory

## Abstract

Hard C-means (HCM; k-means) is one of the most widely used partitive clustering techniques. However, HCM is strongly affected by noise objects and cannot represent cluster overlap. To reduce the influence of noise objects, objects distant from cluster centers are rejected in some noise rejection approaches including general noise rejection (GNR) and cluster-wise noise rejection (CNR). Generalized rough C-means (GRCM) can deal with positive, negative, and boundary belonging of object to clusters by reference to rough set theory. GRCM realizes cluster overlap by the linear function threshold-based object-cluster assignment. In this study, as a unified approach for GNR and CNR in HCM, we propose linear function threshold-based C-means (LiFTCM) by relaxing GRCM. We show that the linear function threshold-based assignment in LiFTCM includes GNR, CNR, and their combinations as well as rough assignment of GRCM. The classification boundary is visualized so that the characteristics of LiFTCM in various parameter settings are clarified. Numerical experiments demonstrate that the combinations of rough clustering or the combinations of GNR and CNR realized by LiFTCM yield satisfactory results.

## Introduction

Clustering, which is an important task in data mining/machine learning, is a technique for automatically extracting group (cluster) structures from data without supervision. It is useful for analyzing large-scale unlabeled data. Hard *C*-means (HCM; *k*-means) (MacQueen, 1967) is one of the most widely used partitive clustering techniques. Real-world datasets often contain noise objects (outliers) with irregular features that may distort cluster shapes and deteriorate clustering performance. Since *C*-means-type methods are formulated based on the minimization of the total within-cluster sum-of-squared-error, they are strongly affected by noise objects, which are distant from cluster centers. We focus on two types of noise rejection, namely, general noise rejection (GNR) and cluster-wise noise rejection (CNR). In GNR approaches, whether each object is noise or not is defined in the whole cluster structure. Objects distant from any cluster center are rejected as noise. On the other hand, in CNR approaches, whether each object is noise or not is defined for each cluster. For each cluster, objects distant from its center are rejected as noise. Both GNR and CNR perform noise rejection while GNR performs exclusive cluster assignment whereas CNR allows cluster overlap.

HCM assigns each object to one and only one cluster with membership in the Boolean (hard; crisp) domain {0, 1}, and thus it cannot represent belonging to multiple clusters or non-belonging to any cluster. However, in real-world datasets, belonging of object to clusters is often unclear. Soft computing approaches are useful to represent belonging to multiple clusters or non-belonging to any cluster. Clustering based on rough set theory (Pawlak, 1982; Pawlak, 1991) considers positive, negative, and boundary belonging of object to clusters. Lingras and West proposed rough *C*-means (LRCM) (Lingras & West, 2004) as a rough-set-based *C*-means clustering, and Peters proposed a refined version of RCM (PRCM) (Peters, 2006). Ubukata et al. proposed the generalized RCM (GRCM) (Ubukata, Notsu & Honda, 2017) by integrating LRCM and PRCM. GRCM realizes cluster overlap by a linear function threshold with respect to the distance to the nearest cluster and detects the upper area composed of objects that possibly belong to the cluster. Specifically, the threshold based on the distance to the nearest cluster center is lifted by the linear function to allow the cluster to be assigned to relatively near clusters as well as the nearest cluster.

In this study, we investigate the characteristics of the linear function threshold-based object-cluster assignment in GRCM. We show that the linear function threshold-based assignment in relaxed GRCM can realize GNR, CNR, and their combinations as well as rough assignments. One important point is that the linear function threshold-based assignment essentially includes GNR and CNR in compliance with RCM standards without any extra formulation. As a unified approach for GNR and CNR in HCM, we propose linear function threshold-based *C*-means (LiFTCM) by relaxing GRCM. The classification boundary is visualized so that the characteristics of LiFTCM in various parameter settings are clarified. Numerical experiments demonstrate that the combinations of rough clustering or the combinations of GNR and CNR realized by LiFTCM yield satisfactory results.

The remainder of the paper is organized as follows. In “Related Work,” related works are discussed. “Preliminaries” presents the preliminaries for clustering methods. In “A unified approach for cluster-wise and general noise rejection approaches,” we show that the linear function threshold-based assignment in relaxed GRCM can realize GNR, CNR, and their combinations as well as rough assignments. In “Proposed Method,” we propose LiFTCM as one of the relaxed GRCM. In “Visualization of Classification Boundaries,” the classification boundaries of LiFTCM with various parameter settings are considered. In “Numerical Experiments,” the clustering performance of LiFTCM with various parameter settings is discussed. In “Discussion,” the calculation of the cluster center in the proposed method are discussed. Finally, the conclusions are presented in “Conclusions.”

## Related Work

### Noise rejection in regression analysis and *C*-means-type clustering

Many machine learning tasks such as regression analysis are formulated in a framework of least mean squares (LMS) proposed by Legendre or Gauss (Legendre, 1805; Gauss, 1809), which minimizes the sum of the squared residuals to fit models to a dataset. However, since the LMS criterion is strongly affected by noise objects and has the lack of robustness, various robust estimation methods have been proposed to reduce the influence of noise objects. Least absolute values (LAV) (Edgeworth, 1887) is a criterion that minimizes the sum of the absolute values of the residuals to reduce the influence of large residuals. M-estimator (Huber, 1964; Huber, 1981) is one of the most widely used robust estimators, which replaces the square function in LMS by a symmetric function with a unique minimum at zero that reduces the influence of large residuals. Least median of squares (LMedS) (Hampel, 1975; Rousseeuw, 1984) minimizes the median of the squared residuals. Least trimmed squares (LTS) (Rousseeuw & Leroy, 1987) minimizes the sum of the squared residuals up to *h*-th objects in ascending list of residuals.

Since *C*-means-type clustering methods are generally formulated based on the minimization of the within-cluster sum-of-squared-error, the above-mentioned robust estimation methods are promising approaches to noise in the cluster structure (Kaufmann & Rousseeuw, 1987; Dubes & Jain, 1988). In *C*-means-type clustering, the distance between object and its nearest cluster center is identified as the residual. Thus, in GNR, objects distant from any cluster center are rejected as noise. For instance, trimmed *C*-means (TCM; trimmed *k*-means, TKM) (Cuesta-Albertos, Gordaliza & Matrán, 1997; Garcia-Escudero & Gordaliza, 1999) introduces LTS criterion to HCM. TCM calculates the new cluster center by using objects up to *h*-th in ascending list of the distances to their nearest cluster centers. As a result, objects more than a certain distance away are rejected as noise. Noise rejection in *C*-means-type clustering is also well discussed in the context of fuzzy *C*-means (FCM) (Dunn, 1973; Bezdek, 1981). In noise fuzzy *C*-means (NFCM) (Davé, 1991; Davé & Krishnapuram, 1997), a single noise cluster is introduced in addition to the intended regular clusters and objects distant from any cluster center are absorbed in the noise cluster. Another approach to noise is CNR. For instance, possibilistic *C*-means (PCM) (Krishnapuram & Keller, 1993; Krishnapuram & Keller, 1996) considers cluster-wise noise rejection, in which each cluster is extracted independently while rejecting objects distant from its center. The membership values are interpreted as degrees of possibility of the object belonging to clusters. PCM represents typicality as absolute membership to clusters rather than relative membership by eliminating the sum-to-one constraint. Fuzzy possibilistic *C*-means (FPCM) (Pal, Pal & Bezdek, 1997) uses both relative typicalities (memberships) and absolute typicalities. Possibilistic fuzzy *C*-means (PFCM) (Pal et al., 2005) is a hybridization of FCM and PCM using both probabilistic memberships of FCM and possibilistic memberships of PCM.

In this study, we show that GNR, CNR, and their combinations are realized by the linear function threshold-based object-cluster assignment in the proposed LiFTCM. The above-mentioned approaches introduce various mechanisms to realize GNR and CNR. In contrast, the linear function threshold-based assignment essentially includes GNR, CNR, and their combinations in compliance with RCM standards without any extra formulation.

### Generalized approaches to hard, fuzzy, noise, possibilistic, and rough clustering

Maji & Pal (2007a) proposed rough-fuzzy *C*-means (RFCM) as a hybrid algorithm of FCM and RCM. RFCM is formulated so that objects in the lower areas have crisp memberships and objects in the boundary areas have FCM-based fuzzy memberships. Furthermore, Maji & Pal (2007b) proposed rough-fuzzy possibilistic *C*-means (RFPCM)  based on possibilistic fuzzy *C*-means (PFCM) (Pal et al., 2005). Masson and Denœux proposed evidential *C*-means (ECM) (Masson & Denoeux, 2008) as one of the evidential clustering (EVCLUS) (Denoeux & Masson, 2003; Denoeux & Masson, 2004) methods based on the Dempster-Shafer theory of belief functions (evidence theory). Evidential clustering considers the basic belief assignment, which indicates the membership (mass of belief) of each object to each subset of clusters with the probabilistic constraints that derive *credal* partition. Credal partition can represent hard and fuzzy partitions with a noise cluster considering assignments to a singleton and the empty set. Possibilistic and rough partitions are represented by using the plausibility function and the belief function (Denoeux & Kanjanatarakul, 2016).

Although RFCM and RFPCM provide interesting perspectives on the handling of the uncertainty in the boundary area, the object-cluster assignment is different from that of RCM and transform into different types of approach. Although the credal partition in ECM has high expressiveness including hard, noise, possibilistic, and rough clustering, the object-cluster assignment and cluster center calculation of ECM do not boil down to those of RCM. In contrast to the above-mentioned approaches, the formulation of the proposed LiFTCM is fully compliant with RCM standards. This study reveals that RCM itself inherently includes GNR, CNR, and their combinations as well as rough clustering aspects without any extra formulation.

## Preliminaries

### Hard *C*-means and noise rejection

Let *U* = {***x***_1_, …, ***x***_*i*_, …, ***x***_*n*_} be a set of *n* objects, where each object ***x***_*i*_ = (*x*_*i*1_, …, *x*_*ij*_, …, *x*_*ip*_)^⊤^ is a *p*-dimensional real feature vector. In *C*-means-type methods, *C*(2 ≤ *C* < *n*) represents the number of clusters, and *C* clusters composed of similar objects are extracted from *U*. Each cluster has a prototypical point (cluster center), which is a *p*-dimensional vector ***b***_*c*_ = (*b*_*c*1_, …, *b*_*cj*_, …, *b*_*cp*_)^⊤^. Let *u*_*ci*_ be the degree of belonging of object *i* to cluster *c*. Let *d*_*ci*_ = ||***x***_*i*_ − ***b***_*c*_|| be the distance between the cluster center ***b***_*c*_ and the object *i*.

The optimization problem of HCM (MacQueen, 1967) is given by

(1) \begin{eqnarray*}& & \text{min.}\quad {J}_{HCM}=\sum _{c=1}^{C}\sum _{i=1}^{n}{u}_{ci}{d}_{ci}^{2},\end{eqnarray*}

(2) \begin{eqnarray*}& & \text{s.t.}\quad {u}_{ci}\in \{0,1\},\forall c,i,\end{eqnarray*}

(3) \begin{eqnarray*}& & \sum _{c=1}^{C}{u}_{ci}=1,\forall i.\end{eqnarray*}

HCM minimizes the total within-cluster sum-of-squared-error (Eq. (1)) under the Boolean domain constraints (Eq. (2)) and the sum-to-one constraints across clusters (Eq. (3)).

HCM first initializes cluster centers and then alternately updates *u*_*ci*_ and ***b***_*c*_ until convergence by using the following update rules:

(4) \begin{eqnarray*}& & {u}_{ci}= \left\{ \begin{array}{@{}ll@{}} \displaystyle 1 &\displaystyle \left( c=\argmin _{1\leq l\leq C}{d}_{li} \right) ,\\ \displaystyle 0 &\displaystyle (\text{otherwise}), \end{array} \right. \end{eqnarray*}

(5) \begin{eqnarray*}& & {\mathbi{b}}_{c}= \frac{\sum _{i=1}^{n}{u}_{ci}{\mathbi{x}}_{i}}{\sum _{i=1}^{n}{u}_{ci}} .\end{eqnarray*}

There are various strategies for initializing cluster centers. A naive strategy is to choose *C* objects as initial cluster centers from *U* by simple random sampling without replacement. Alternatively, there are strategies that set the initial cluster centers away from each other to reduce initial value dependencies and improve clustering performance, such as KKZ (Katsavounidis, Kuo & Zhang, 1994) and *k*-means++ (Arthur & Vassilvitskii, 2007).

#### General noise rejection (GNR)

Since HCM is formulated based on the LMS criterion, it is strongly affected by noise objects. Like TCM, which introduces the LTS criterion, the influence of noise objects can be reduced by rejecting objects distant from any cluster. In this type of GNR, each object is assigned to the nearest cluster under the condition that the distance *d*_*ci*_ is less than or equal to a threshold (noise distance) *δ*(*δ* > 0):

(6) \begin{eqnarray*}{u}_{ci}= \left\{ \begin{array}{@{}ll@{}} \displaystyle 1 &\displaystyle \left( c=\argmin _{1\leq l\leq C}{d}_{li}\wedge {d}_{ci}\leq \delta \right) ,\\ \displaystyle 0 &\displaystyle (\text{otherwise}). \end{array} \right. \end{eqnarray*}

The smaller *δ* is, the more objects are rejected as noise. The noise distance *δ* can depend on how many (what percentage of) objects to reject as noise.

#### Cluster-wise noise rejection (CNR)

GNR is based on HCM-based exclusive assignment and cannot represent cluster overlap. By performing noise rejection independently for each cluster, possibilistic aspects that present non-belonging to any cluster and belonging to multiple clusters are achieved. In this type of CNR, noise rejection is performed for each cluster by rejecting objects over *δ*_*c*_ distant from its center:

(7) \begin{eqnarray*}{u}_{ci}= \left\{ \begin{array}{@{}ll@{}} \displaystyle 1 &\displaystyle ({d}_{ci}\leq {\delta }_{c}),\\ \displaystyle 0 &\displaystyle (\text{otherwise}). \end{array} \right. \end{eqnarray*}

The smaller *δ*_*c*_ is, the more objects are rejected as noise for each cluster *c*. The cluster-wise noise distance *δ*_*c*_ can depend on how many (what percentage of) objects to reject as noise for each cluster.

### Generalized rough *C*-means

In RCM-type methods, which are rough set clustering schemes, membership in the lower, upper, and boundary areas of each cluster represents positive, possible, and uncertain belonging to the cluster, respectively (Lingras & West, 2004; Peters, 2006; Peters et al., 2013; Ubukata, Notsu & Honda, 2017). GRCM is constructed based on a heuristic scheme, not an objective function.

**Figure 1:**
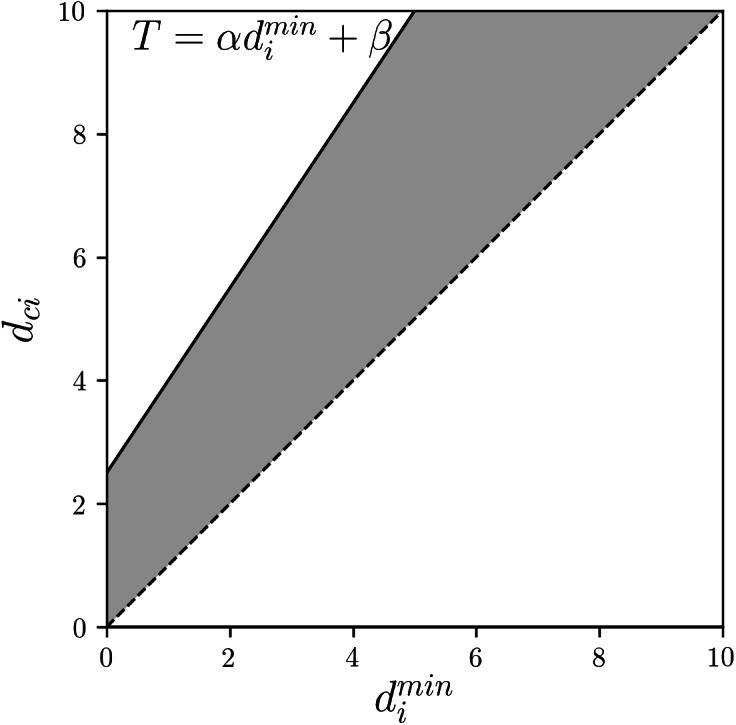
GRCM: the linear function threshold *T* and the allowable range of *d*_*ci*_ (gray area).

In every iteration, the membership ${\overline{u}}_{ci}$ of object *i* to the upper area of cluster *c* is first calculated as follows:

(8) \begin{eqnarray*}& & {d}_{i}^{\min \nolimits }=\min _{1\leq l\leq C}{d}_{li},\end{eqnarray*}

(9) \begin{eqnarray*}& & {\overline{u}}_{ci}= \left\{ \begin{array}{@{}l@{}} \displaystyle 1 ({d}_{ci}\leq \alpha {d}_{i}^{\min \nolimits }+\beta ), \\ \displaystyle 0 (\text{otherwise}), \end{array} \right. \end{eqnarray*}

where *α* (*α* ≥ 1) and *β* (*β* ≥ 0) are user-defined parameters that adjust the volume of the upper areas. GRCM assigns each object to the upper area of not only its nearest cluster but also of other relatively nearby clusters using a linear function of the distance to its nearest cluster as a threshold. Larger *α* and *β* imply larger clustering roughness and larger overlap of the upper areas of the clusters. Figure 1 shows the linear function threshold *T* and the allowable range of *d*_*ci*_ (gray area) in GRCM.

The membership ${\underline{u}}_{ci}$ and ${\hat {u}}_{ci}$ of object *i* to the lower and boundary areas, respectively, is calculated using ${\overline{u}}_{ci}$ as follows:

(10) \begin{eqnarray*}& & {\underline{u}}_{ci}= \left\{ \begin{array}{@{}l@{}} \displaystyle 1 \left( {\overline{u}}_{ci}=1\wedge \sum _{l=1}^{C}{\overline{u}}_{li}=1 \right) , \\ \displaystyle 0 (\text{otherwise}), \end{array} \right. \end{eqnarray*}

(11) \begin{eqnarray*}& & {\hat {u}}_{ci}= \left\{ \begin{array}{@{}l@{}} \displaystyle 1 \left( {\overline{u}}_{ci}=1\wedge \sum _{l=1}^{C}{\overline{u}}_{li}\not = 1 \right) , \\ \displaystyle 0 (\text{otherwise}) \end{array} \right. \end{eqnarray*}

(12) \begin{eqnarray*}& & ={\overline{u}}_{ci}-{\underline{u}}_{ci}.\end{eqnarray*}

GRCM represents each cluster by the three regions. Therefore, the new cluster center is determined by the aggregation of the centers of these regions. The cluster center ***b***_*c*_ is calculated by the convex combination of the centers of the lower, upper, and boundary areas of the cluster *c*:

(13) \begin{eqnarray*}{\mathbi{b}}_{c}& =& \left\{ \begin{array}{@{}l@{}} \displaystyle \frac{\sum _{i=1}^{n}{\overline{u}}_{ci}{\mathbi{x}}_{i}}{\sum _{i=1}^{n}{\overline{u}}_{ci}} \left( \sum _{i=1}^{n}{\underline{u}}_{ci}=0\vee \sum _{i=1}^{n}{\hat {u}}_{ci}=0 \right) , \\ \displaystyle \underline{w} \frac{\sum _{i=1}^{n}{\underline{u}}_{ci}{\mathbi{x}}_{i}}{\sum _{i=1}^{n}{\underline{u}}_{ci}} +\overline{w} \frac{\sum _{i=1}^{n}{\overline{u}}_{ci}{\mathbi{x}}_{i}}{\sum _{i=1}^{n}{\overline{u}}_{ci}} +\hat {w} \frac{\sum _{i=1}^{n}{\hat {u}}_{ci}{\mathbi{x}}_{i}}{\sum _{i=1}^{n}{\hat {u}}_{ci}} (\text{otherwise}), \end{array} \right. \end{eqnarray*}

(14) \begin{eqnarray*}& & \underline{w},\overline{w},\hat {w}\geq 0,\end{eqnarray*}

(15) \begin{eqnarray*}& & \underline{w}+\overline{w}+\hat {w}=1,\end{eqnarray*}

where $\underline{w}$, $\overline{w}$, and $\hat {w}$ are user-defined parameters that represent the impact of the centers of the lower, upper, and boundary areas, respectively. Ubukata, Notsu & Honda (2017) suggest $\hat {w}=0$ because the centers of the boundary areas tend to cause instability in the calculations and poor classification performance.

## A Unified Approach for Cluster-Wise and General Noise Rejection Approaches

In this section, we show that GNR, CNR, and their combinations are realized by the linear function threshold in relaxed GRCM. Here, we consider relaxing the condition *α* ≥ 1 to *α* ≥ 0 in Eq. (9).

### HCM

In HCM, each object is assigned to the cluster whose center is nearest to the object. This assignment can be interpreted as assigning object *i* to cluster *c* if *d*_*ci*_ is equal to (or less than) ${d}_{i}^{\min }$, that is,

(16) \begin{eqnarray*}{u}_{ci}= \left\{ \begin{array}{@{}l@{}} \displaystyle 1 ({d}_{ci}\leq {d}_{i}^{\min \nolimits }), \\ \displaystyle 0 (\text{otherwise}). \end{array} \right. \end{eqnarray*}

**Figure 2:**
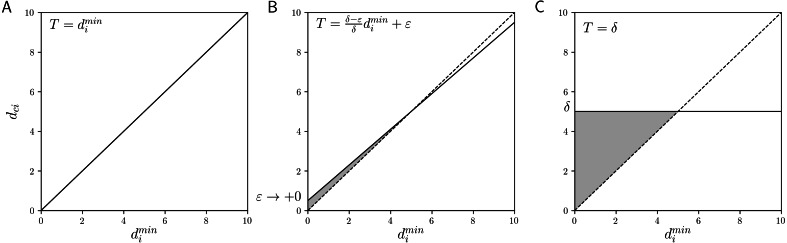
The linear function threshold *T* and the allowable range of *d*_*ci*_ (gray area): (A) HCM, (B) GNR, and (C) CNR.

This is the case *α* = 1 and *β* = 0 in the linear function threshold $\alpha {d}_{i}^{\min }+\beta $ for the assignment of upper area in GRCM (Eq. (9)). Figure 2A shows the linear function threshold *T* and the allowable range of *d*_*ci*_ in HCM. The allowable range is limited to the case ${d}_{ci}={d}_{i}^{\min }$. We note that if there are multiple nearest cluster centers for an object, HCM requires certain tie-breaking rules for satisfying the sum-to-one constraints, such as exclusive assignment based on cluster priority and uniform assignment by distributing the membership, depending on the implementation. However, in the present linear function threshold-based assignment, an object has membership 1 with respect to all its nearest clusters.

The calculation of *u*_*ci*_ in HCM can be represented by that of ${\overline{u}}_{ci}$ in GRCM. The lower and boundary areas are not used in HCM. Thus, the cluster center calculation of HCM is consistent with that of GRCM only using the upper areas, that is, $\overline{w}=1$ in Eq. (13). Therefore, GRCM($\alpha =1,\beta =0,\overline{w}=1$) represents HCM.

### GNR

In GNR, a condition that the distance is less than *δ* is imposed in addition to the threshold-based HCM assignment (Eq. (16)) to reject noise objects over *δ* distant from any clusters. For each object *i* to be assigned to the cluster *c*, *d*_*ci*_ must be equal to (or less than) ${d}_{i}^{\min }$, and equal to or less than the noise distance *δ*, that is,

(17) \begin{eqnarray*}{u}_{ci}= \left\{ \begin{array}{@{}l@{}} \displaystyle 1 ({d}_{ci}\leq {d}_{i}^{\min \nolimits }\wedge {d}_{ci}\leq \delta ), \\ \displaystyle 0 (\text{otherwise}). \end{array} \right. \end{eqnarray*}

This assignment can also be approximated using the linear function threshold by setting $\alpha = \frac{\delta -\varepsilon }{\delta } $ and *β* = ε, where ε → +0, that is,

(18) \begin{eqnarray*}{u}_{ci}= \left\{ \begin{array}{@{}l@{}} \displaystyle 1 \left( {d}_{ci}\leq \frac{\delta -\varepsilon }{\delta } {d}_{i}^{\min \nolimits }+\varepsilon \right) , \\ \displaystyle 0 (\text{otherwise}). \end{array} \right. \end{eqnarray*}

Equation (18) implies that *u*_*ci*_ = 1 if ${d}_{ci}\leq {d}_{i}^{\min }$ and *d*_*ci*_ ≤ *δ*. Thus, Eq. (18) approaches the update rule Eq. (17). In order to show that Eqs. (17) and (18) are equivalent, we show that the condition ${d}_{ci}\leq {d}_{i}^{\min }\wedge {d}_{ci}\leq \delta $ and the condition ${d}_{ci}\leq \frac{\delta -\varepsilon }{\delta } {d}_{i}^{\min }+\varepsilon $ are equivalent, under the condition *δ* > 0 and ε → +0.

Proposition 1 *If* ${d}_{ci}\leq {d}_{i}^{\min }\wedge {d}_{ci}\leq \delta $*, then* ${d}_{ci}\leq \frac{\delta -\varepsilon }{\delta } {d}_{i}^{\min }+\varepsilon $*.*

proof.

(1) ${d}_{ci}\leq {d}_{i}^{\min }\wedge {d}_{ci}\leq \delta $ (Assumption)

(2) ${d}_{ci}\leq {d}_{i}^{\min }$ (Conjunction elimination: (1))

(3) *d*_*ci*_ ≤ *δ* (Conjunction elimination: (1))

(4) ${d}_{i}^{\min }\leq {d}_{ci}$ (Definition: (Eq. (8)))

(5) ${d}_{i}^{\min }\leq \delta $ (Transitivity: (3), (4))

(6) $ \frac{\varepsilon }{\delta } {d}_{i}^{\min }+ \frac{\delta -\varepsilon }{\delta } {d}_{i}^{\min }\leq \frac{\varepsilon }{\delta } \delta + \frac{\delta -\varepsilon }{\delta } {d}_{i}^{\min }$ (Multiply by $ \frac{\varepsilon }{\delta } $ and add $ \frac{\delta -\varepsilon }{\delta } {d}_{i}^{\min }$ in both sides of (5))

(7) ${d}_{i}^{\min }\leq \frac{\delta -\varepsilon }{\delta } {d}_{i}^{\min }+\varepsilon $ (Deformation: (6))

(8) ${d}_{ci}\leq \frac{\delta -\varepsilon }{\delta } {d}_{i}^{\min }+\varepsilon $ (Transitivity: (2), (7)) □

Proposition 2 *If* ${d}_{ci}\leq \frac{\delta -\varepsilon }{\delta } {d}_{i}^{\min }+\varepsilon $*, then* ${d}_{ci}\leq {d}_{i}^{\min }\wedge {d}_{ci}\leq \delta $*, under the condition that* ε* is sufficiently small.*

proof.

(1) ${d}_{ci}\leq \frac{\delta -\varepsilon }{\delta } {d}_{i}^{\min }+\varepsilon $ (Assumption)

(2) ${d}_{ci}\leq {d}_{i}^{\min }$ (From (1) and ε → +0)

(3) ${d}_{i}^{\min }\leq {d}_{ci}$ (Definition: (Eq. (8)))

(4) ${d}_{ci}\leq \frac{\delta -\varepsilon }{\delta } {d}_{ci}+\varepsilon $ (From (1), (3))

(5) *δd*_*ci*_ ≤ *δd*_*ci*_ − ε*d*_*ci*_ + *δ*ε (Multiply by *δ* in both sides of (4))

(6) *d*_*ci*_ ≤ *δ* (Deformation: (5))

(7) ${d}_{ci}\leq {d}_{i}^{\min }\wedge {d}_{ci}\leq \delta $ (Conjunction introduction: (2), (6)) □

Hence, (Eq. (17)) induces (Eq. (18)), and vice versa.

Figure 2B shows the linear function threshold *T* and the allowable range of *d*_*ci*_ (gray area) in GNR. Since the intersection of the two lines $y= \frac{\delta -\varepsilon }{\delta } {d}_{i}^{\min }+\varepsilon $ and $y={d}_{i}^{\min }$ is (*δ*, *δ*), if *d*_*ci*_ > *δ*, object *i* is never assigned to cluster *c*. If ${d}_{i}^{\min }\leq \delta $, the threshold approaches the HCM-based nearest assignment. These characteristics are consistent with those of GNR.

Similar to HCM, in GNR, the cluster centers are calculated only using the upper areas. Therefore, GRCM($\alpha = \frac{\delta -\varepsilon }{\delta } ,\beta =\varepsilon ,\overline{w}=1$) represents GNR.

#### CNR

The object-cluster assignment of CNR is determined only by the magnitude relation between *d*_*ci*_ and *δ*_*c*_ without considering ${d}_{i}^{\min }$. We note that the case *α* = 0 and *β* = *δ*_*c*_ in Eq. (9) corresponds to the update rule Eq. (7) of CNR. Figure 2C shows the linear function threshold *T* and the allowable range of *d*_*ci*_ (gray area) in CNR. Independent of ${d}_{i}^{\min }$, if *d*_*ci*_ ≤ *δ*, object *i* is assigned to cluster *c*.

Similar to HCM and GNR, in CNR, the cluster centers are calculated only using the upper areas. Therefore, GRCM($\alpha =0,\beta ={\delta }_{c},\overline{w}=1$) represents CNR.

### Smooth transition between GNR and CNR by tuning linear function threshold

In reference to the threshold-based assignment of GNR, i.e., Eq. (18), we construct the following rule using a parameter *t* ∈ [0, *δ*_*c*_]:

(19) \begin{eqnarray*}{u}_{ci}= \left\{ \begin{array}{@{}l@{}} \displaystyle 1 ({d}_{ci}\leq \frac{{\delta }_{c}-t}{{\delta }_{c}} {d}_{i}^{\min \nolimits }+t), \\ \displaystyle 0 (\text{otherwise}). \end{array} \right. \end{eqnarray*}

If *t* = 0, then Eq. (19) reduces to Eq. (16) of HCM. If *t* = ε, where ε → +0, then Eq. (19) changes to Eq. (18) of GNR. If *t* = *δ*_*c*_, then Eq. (19) reduces to Eq. (7) of CNR. If *t* ∈ (0, *δ*_*c*_), then Eq. (19) represents the combinations of GNR and CNR. Thereby, smooth transition between HCM, GNR, and CNR is realized.

**Figure 3:**
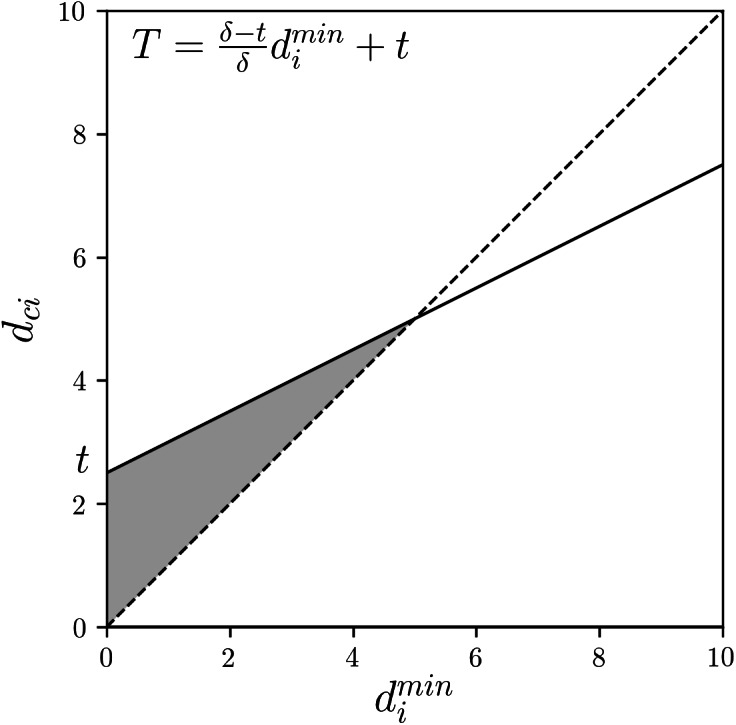
Combination of GNR and CNR: the linear function threshold *T* and the allowable range of *d*_*ci*_ (gray area).

Figure 3 shows the linear function threshold *T* and the allowable range of *d*_*ci*_ (gray area) in the combinations of GNR and CNR. It can be seen that this linear function can transition between the states shown in Fig. 2 by *t*.

For practical use, we consider the normalized parameter *z* ∈ [0, 1]. We let $z= \frac{t}{{\delta }_{c}} \in [0,1]$ and replace *t* in Eq. (19) with *zδ*_*c*_:

(20) \begin{eqnarray*}{u}_{ci}= \left\{ \begin{array}{@{}l@{}} \displaystyle 1 ({d}_{ci}\leq (1-z){d}_{i}^{\min \nolimits }+z{\delta }_{c}), \\ \displaystyle 0 (\text{otherwise}). \end{array} \right. \end{eqnarray*}

Then, *z* = 0 represents HCM, *z* → +0 represents GNR, *z* ∈ (0, 1) represents the combinations of GNR and CNR, and *z* = 1 represents CNR. By Eq. (20), the threshold value is represented by the convex combination of ${d}_{i}^{\min }$ and *δ*_*c*_. That is, HCM, GNR, and CNR can be characterized depending on which of ${d}_{i}^{\min }$ and *δ*_*c*_ is emphasized as the threshold value.

## Proposed Method

In this study, we propose LiFTCM as one of the relaxed GRCM. “LiFT” is an acronym that stands for “linear function threshold” and suggests that the threshold is lifted by the linear function.

 
___________________________________________________________________________________________________ 
Algorithm 1 LiFTCM 
___________________________________________________________________________________________________ 
  Step 1.  Determine α (α ≥ 0),  β (β  ≥ 0),  and w ,__w, ˆ w ≥ 0 such that w  + __w + ˆ w = 1. 
  Step 2. Initialize bc. 
  Step 3. Calculate __uci using Eqs. (8) and (9). 
  Step 4. Calculate u ci and ˆ uci using Eqs. (10) and (11). 
  Step 5. Calculate bc using Eq. (13). 
  Step 6. Repeat Steps 3-5 until __uci do not change. 
___________________________________________________________________________________________________    

A sample procedure of LiFTCM is described in algorithm 1.

Although this algorithm just corresponds to the case where the condition *α* ≥ 1 in GRCM is relaxed to *α* ≥ 0, LiFTCM can represent GNR, CNR, and their combinations in addition to GRCM. If 0 ≤ *α* ≤ 1, LiFTCM includes HCM, GNR, CNR, and their combinations. If *α* ≥ 1, LiFTCM includes HCM, LRCM, PRCM, and their combinations. Table 1 summarizes the relationships between HCM, GNR, CNR, and rough clustering, and their combinations depending on the values of the parameters *α* and *β* in LiFTCM.

**Table 1:** Relationship between HCM, GNR, CNR, and rough clustering, and their combinations in terms of the linear function threshold in LiFTCM.

Linear function Threshold: $\alpha {d}_{i}^{\min }+\beta $	*β* = 0	*β* → +0	0 < *β*
*α* = 0	–	–	CNR
0 < *α* < 1	–	GNR	Combinations of GNR and CNR
*α* = 1	HCM	HCM	LRCM
1 < *α*	PRCM	PRCM	Combinations of LRCM and PRCM (GRCM)

As it is difficult to adjust noise sensitivity by directly changing *α* and *β* when noise rejection is considered in LiFTCM, it is convenient to fix the cluster-wise noise distance *δ*_*c*_ and adjust the combination of HCM, GNR, and CNR by the parameter *z* ∈ [0, 1] with *α* = (1 − *z*) and *β* = *zδ*_*c*_.

The representations of the conventional methods by setting the parameters of LiFTCM are summarized as follows:

1. HCM: LiFTCM(*α* = 1, *β* = 0, $\overline{w}=1$).
2. LRCM: LiFTCM(*α* = 1, *β* ≥ 0, $\overline{w}=0$).
3. PRCM: LiFTCM(*α* ≥ 1, *β* = 0, $\hat {w}=0$).
4. GRCM: LiFTCM(*α* ≥ 1, *β* ≥ 0).
5. GNR: LiFTCM(*α* = 1 − *z*, *β* = *zδ*_*c*_, *z* → +0, $\overline{w}=1$).
6. CNR: LiFTCM(*α* = 0, *β* = *δ*_*c*_, $\overline{w}=1$).
7. Combinations of GNR and CNR: LiFTCM(*α* = 1 − *z*, *β* = *zδ*_*c*_, *z* ∈ [0, 1], $\overline{w}=1$).

## Visualization of Classification Boundaries

In this section, we visualize the classification boundaries of the proposed LiFTCM. LiFTCM was applied to a grid point dataset, in which *n* = 100 × 100 objects are uniformly arranged in the unit square [0, 1] × [0, 1]. *C* = 3 clusters (*c* = 1, 2, 3), which correspond to the primary colors (*Red*, *Green*, *Blue*), respectively, are extracted by LiFTCM. The RGB-color of object *i* is determined by $(R,G,B)_{i}=(255\times {\overline{u}}_{1i},255\times {\overline{u}}_{2i},255\times {\overline{u}}_{3i})$. Objects belonging to a single cluster are represented by primary colors, objects belonging to multiple clusters are represented by additive colors, and objects not belonging to any cluster are represented by black color. The cluster centers are indicated by cross marks. Initial cluster centers were determined by ***b***_1_ = (0, 0)^⊤^, ***b***_2_ = (0.5, 1)^⊤^, and ***b***_3_ = (1, 0)^⊤^.

**Figure 4:**
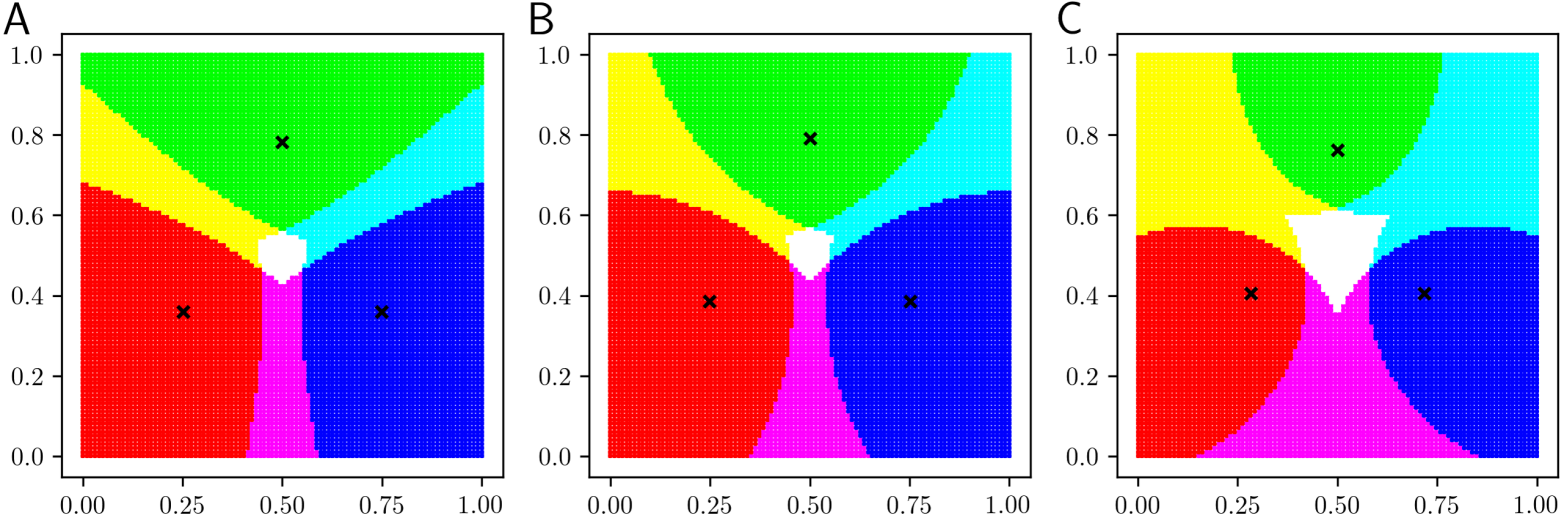
Classification boundaries of LiFTCM(*α* ≥ 1, *β* ≥ 0, $\overline{w}=1$) representing LRCM, PRCM, and GRCM assignments: (A) LiFTCM(*α* = 1, *β* = 0.1, $\overline{w}=1$) (LRCM assignment), (B) LiFTCM(*α* = 1.4, *β* = 0, $\overline{w}=1$) (PRCM assignment), and (C) LiFTCM(*α* = 1.4, *β* = 0.1, $\overline{w}=1$) (GRCM assignment).

Figure 4 shows the results of LiFTCM(*α* ≥ 1, *β* ≥ 0, $\overline{w}=1$), which corresponds to GRCM($\overline{w}=1$). Figure 4A shows the result of LiFTCM(*α* = 1, *β* = 0.1, $\overline{w}=1$), which is interpreted as the LRCM assignment. Figure 4B shows the result of LiFTCM(*α* = 1.4, *β* = 0, $\overline{w}=1$), which is interpreted as the PRCM assignment. Figure 4C shows the result of LiFTCM(*α* = 1.4, *β* = 0.1, $\overline{w}=1$), which is interpreted as the GRCM assignment. Thereby, cluster overlap is realized by lifting the threshold by a linear function.

**Figure 5:**
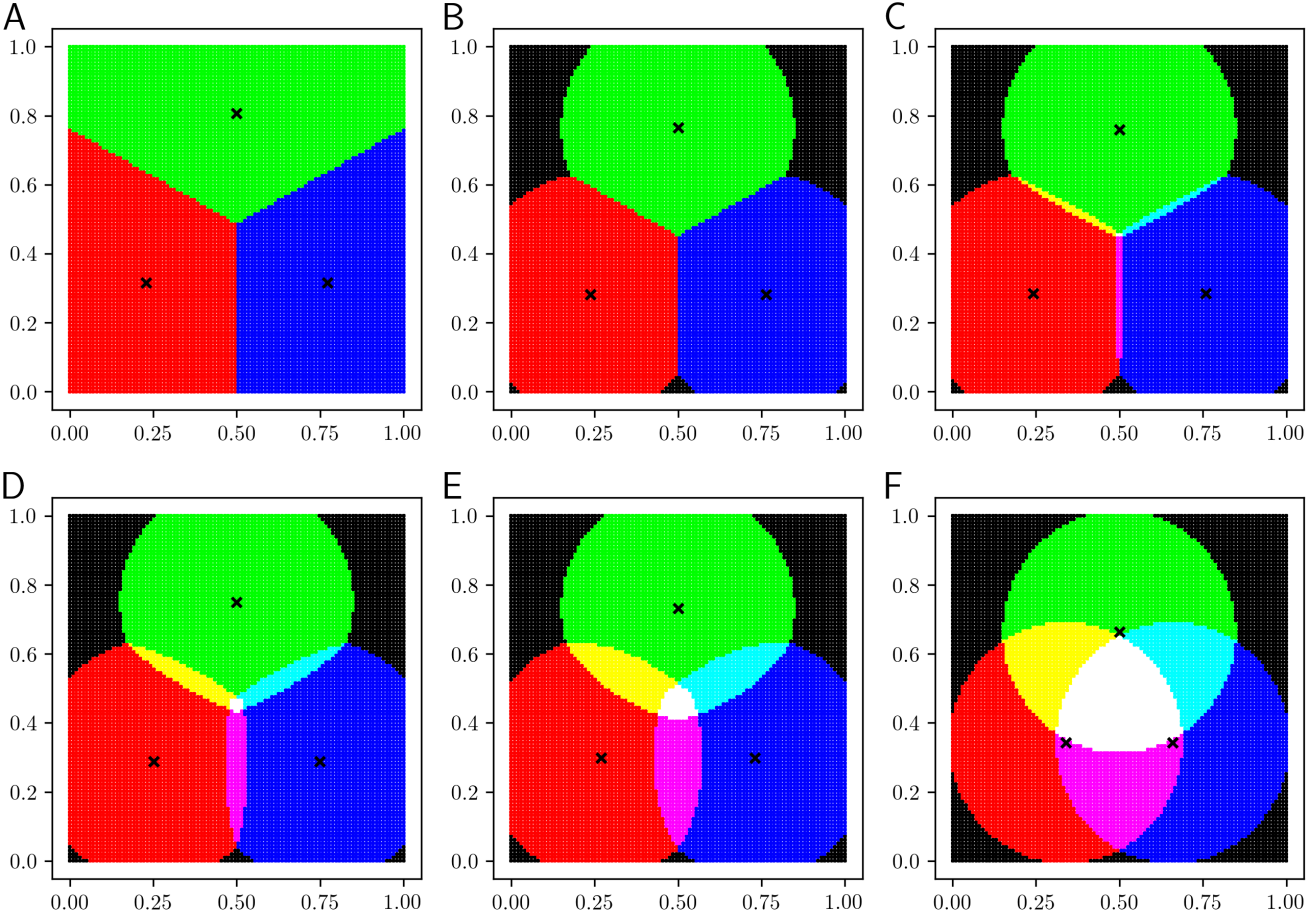
Classification boundaries of LiFTCM(*α* = 1 − *z*, *β* = *zδ*_*c*_, $\overline{w}=1$) representing HCM, GNR, CNR, and their combinations: (A) *z* = 0 (HCM), (B) *z* = 0.001 (GNR), (C) *z* = 0.25 (combination), (D) *z* = 0.5 (combination), (E) *z* = 0.75 (combination), and (F) *z* = 1 (CNR).

Figure 5 shows the results of LiFTCM(*α* = 1 − *z*, *β* = *zδ*_*c*_, *z* ∈ [0, 1], $\overline{w}=1$) in which noise rejection is intended. The noise distance was set to *δ*_*c*_ = 0.35 and the parameter *z* was set to {0, 0.001, 0.25, 0.5, 0.75, 1}. Figure 5A shows the result for *z* = 0. A hard partition with a Voronoi boundary is generated in the same manner as in HCM. Figure 5B shows the result for *z* = 0.001. Such a small value of *z* realize general noise rejection, that is, objects over *δ*_*c*_ distant from any cluster are rejected. The boundary between clusters is the Voronoi boundary, and objects whose distance to any cluster is greater than the noise distance *δ*_*c*_ are shown in black and rejected as noise. As *z* approaches 1 in the order of Figs. 5C–5E, the overlap between clusters increases. Figure 5F shows the result for *z* = 1. In this case, cluster-wise noise rejection is performed and each cluster is composed of a circle with radius *δ*_*c*_ centered at the cluster center. By adjusting the threshold relative to *δ*_*c*_, cluster overlap and noise rejection are realized simultaneously.

Thereby, LiFTCM can realize HCM, GRCM, GNR, CNR, and their combinations by lifting the threshold by a linear function.

### Schematic diagram

**Figure 6:**
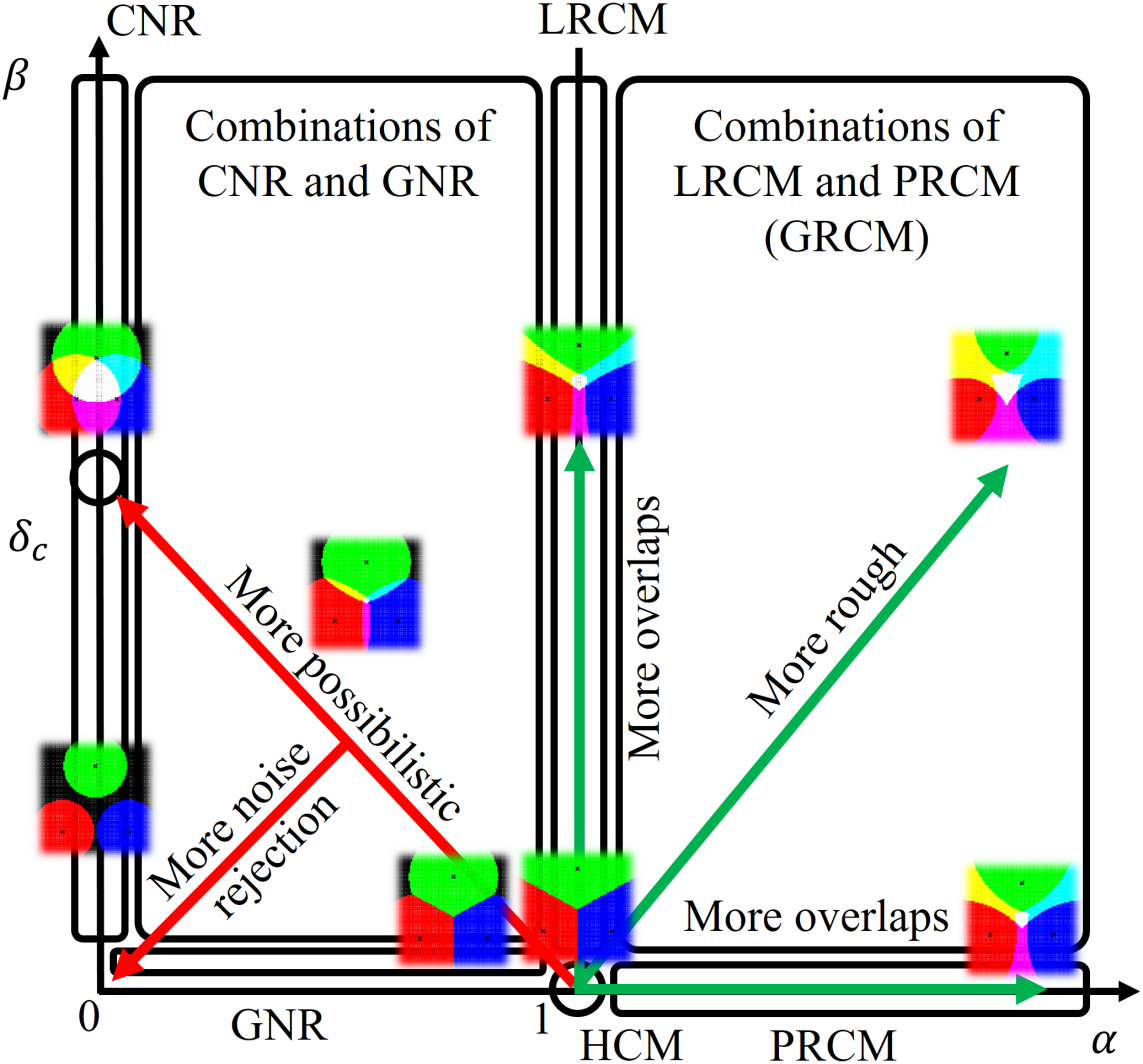
Schematic diagram: representations of HCM, LRCM, PRCM, GRCM, GNR, CNR, and their combinations by a linear function threshold in LiFTCM with the parameters (*α*, *β*), and their relationships.

Figure 6 is a schematic diagram of the proposal of this study. Representations of HCM, LRCM, PRCM, GRCM, GNR, CNR, and their combinations by the linear function threshold in LiFTCM with the parameters (*α*, *β*), and their relationships are shown. (*α*, *β*) = (1, 0) is the default state and represents HCM assignment. Increasing *α* from 1 and *β* from 0 increases cluster overlap. Simultaneously increasing *α* and *β* increases clustering roughness. This shows combinations of LRCM and PRCM, namely, GRCM. LiFTCM gives an interpretation in 0 ≤ *α* ≤ 1 in addition to GRCM. As proposed in the smooth transition, when the parameter *z* is increased from 0 to 1, (*α*, *β*) transits from (1, 0) to (0, *δ*_*c*_), namely, from HCM to CNR via GNR. The parameter *z* has the effect of changing clustering more possibilistic. Cluster overlap in CNR is attributed to the increase in *β* in LRCM. The direction in which the destination *δ*_*c*_ is lowered is the direction in which noise objects are more rejected.

## Numerical Experiments

This section presents the results of numerical experiments for evaluating the clustering performance of the proposed LiFTCM with various parameter settings in four real-world datasets downloaded from UCI Machine Learning Repository (https://archive.ics.uci.edu/ml/) and summarized in Table 2. Performance was evaluated by the accuracy of class center estimation. The datasets are labeled and include the feature vector and the correct class label of each object. Each dataset was partitioned into disjoint classes according to the class labels, and the center of each class (class center) was calculated. LiFTCM was applied to the generated unlabeled datasets. The number *C* of clusters was set to the number of classes. To avoid initial value dependence, the initial cluster centers were set to the cluster centers generated by KKZ-based HCM. Considering the correspondence of the clusters and the classes, the minimum total error of the cluster centers and the class centers, which is called center-error, was taken as the measurement value. Let ${\hat {\mathbi{b}}}_{c}$ be the class center of the class corresponding to cluster *c*. Center-error is calculated by

(21) \begin{eqnarray*}center\text{_}error=\sum _{c=1}^{C}{|}{|}{\mathbi{b}}_{c}-{\hat {\mathbi{b}}}_{c}{|}{|}.\end{eqnarray*}

If the center-error is small, the accuracy of class center estimation is high, and clustering performance is assumed to be high.

**Table 2:** Characteristics of the datasets and the range of parameters *α*, *β*, *δ*_*c*_, and *z*, which tune the linear function threshold in LiFTCM.

Dataset	#classes	#features	#objects (#objects in classes)	Settings of parameters
Iris	3	4	150 (50, 50, 50)	*α* ∈ [1, 1.2], *β* ∈ [0, 0.6], *δ*_*c*_ ∈ [0.85, 1.5], *z* ∈ [0, 1]
Wine	3	13	178 (59, 71, 48)	*α* ∈ [1, 2.4], *β* ∈ [0, 250], *δ*_*c*_ ∈ [150, 1000], *z* ∈ [0, 1]
Glass	6	9	214 (70, 76, 17, 13, 9, 29)	*α* ∈ [1, 1.6], *β* ∈ [0, 1.5], *δ*_*c*_ ∈ [10, 30], *z* ∈ [0, 1]
Breast Cancer Wisconsin	2	9	683 (444, 239)	*α* ∈ [1, 1.5], *β* ∈ [0, 4], *δ*_*c*_ ∈ [5, 70], *z* ∈ [0, 1]

**Figure 7:**
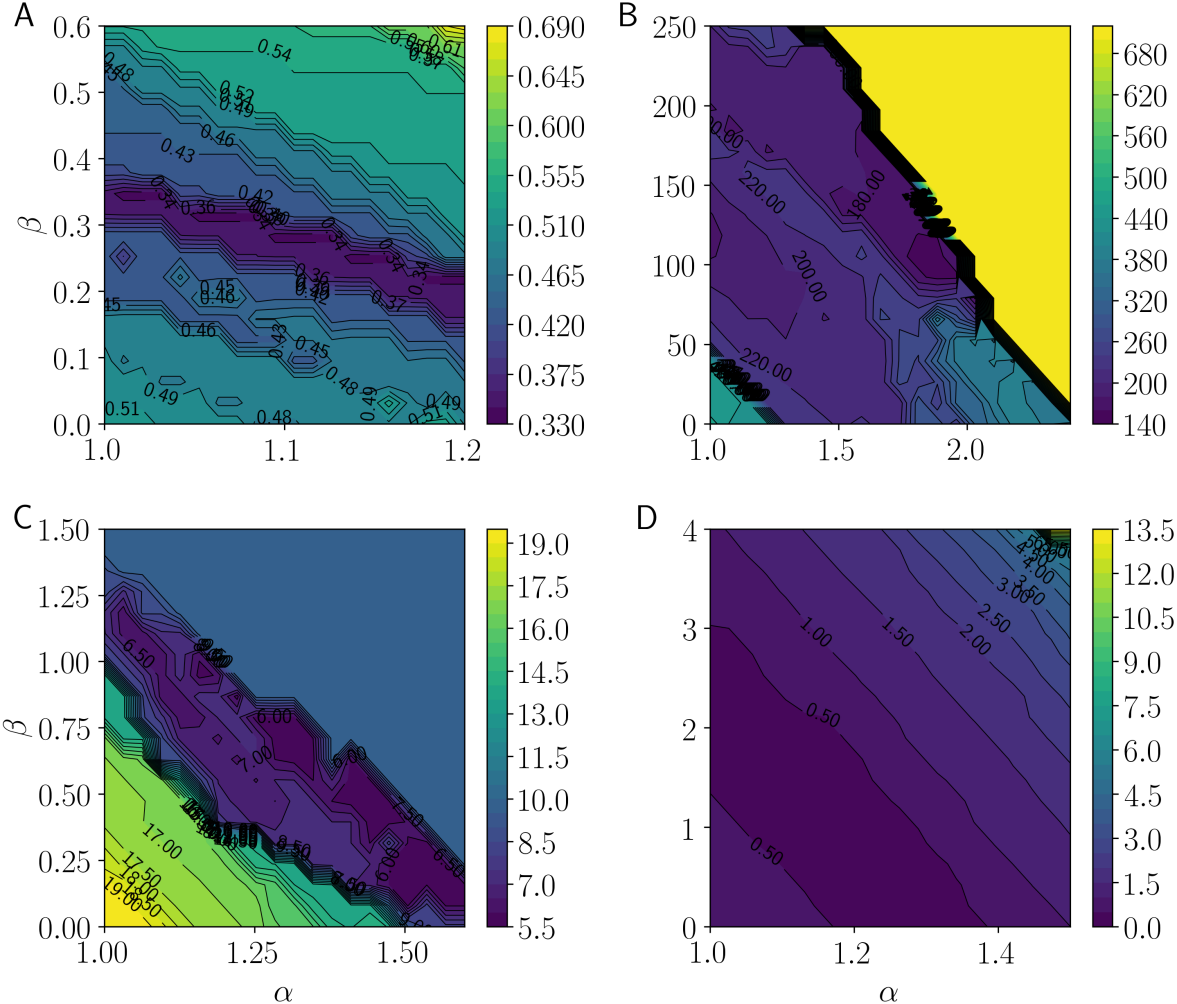
Minimum total errors between cluster centers and class centers by LiFTCM(*α* ≥ 1, *β* ≥ 0, $\overline{w}=1$) representing GRCM($\overline{w}=1$): (A) Iris, (B) Wine, (C) Glass, and (D) Breast Cancer Wisconsin.

Figure 7 shows the center-error measurements as *α* and *β* take 100 equally distributed values using contour lines. Colors closer to purple imply smaller center-error and hence better clustering performance. Figure 7A shows the results for the Iris dataset. Performance is improved at approximately *α* = 1 and *β* = 0.35, and when *α* is increased, performance is maintained by decreasing *β*. This implies that moderate roughness improves performance. Figure 7B shows the results for the Wine dataset. Performance is improved at approximately *α* = 1.9 and *β* = 100. When *α* and *β* exceed certain values, performance deteriorates rapidly. This implies that moderate roughness is acceptable, but excessive roughness degrades performance. Figure 7C shows the results for the Glass dataset. Performance is improved at approximately *α* = 1.3 and *β* = 0.7. As with the Iris dataset, performance is improved with moderate roughness. Figure 7D shows the results for the Breast Cancer Wisconsin dataset. Performance is improved at approximately *α* = 1 and *β* = 2, and it is clear that performance is improved with moderate roughness, as is the case with the Iris and the Glass datasets. Therefore, it is suggested that performance is improved when *α* and *β* are increased to obtain moderate roughness. Thus, the representation of combinations of LRCM and PRCM by LiFTCM performs well.

**Figure 8:**
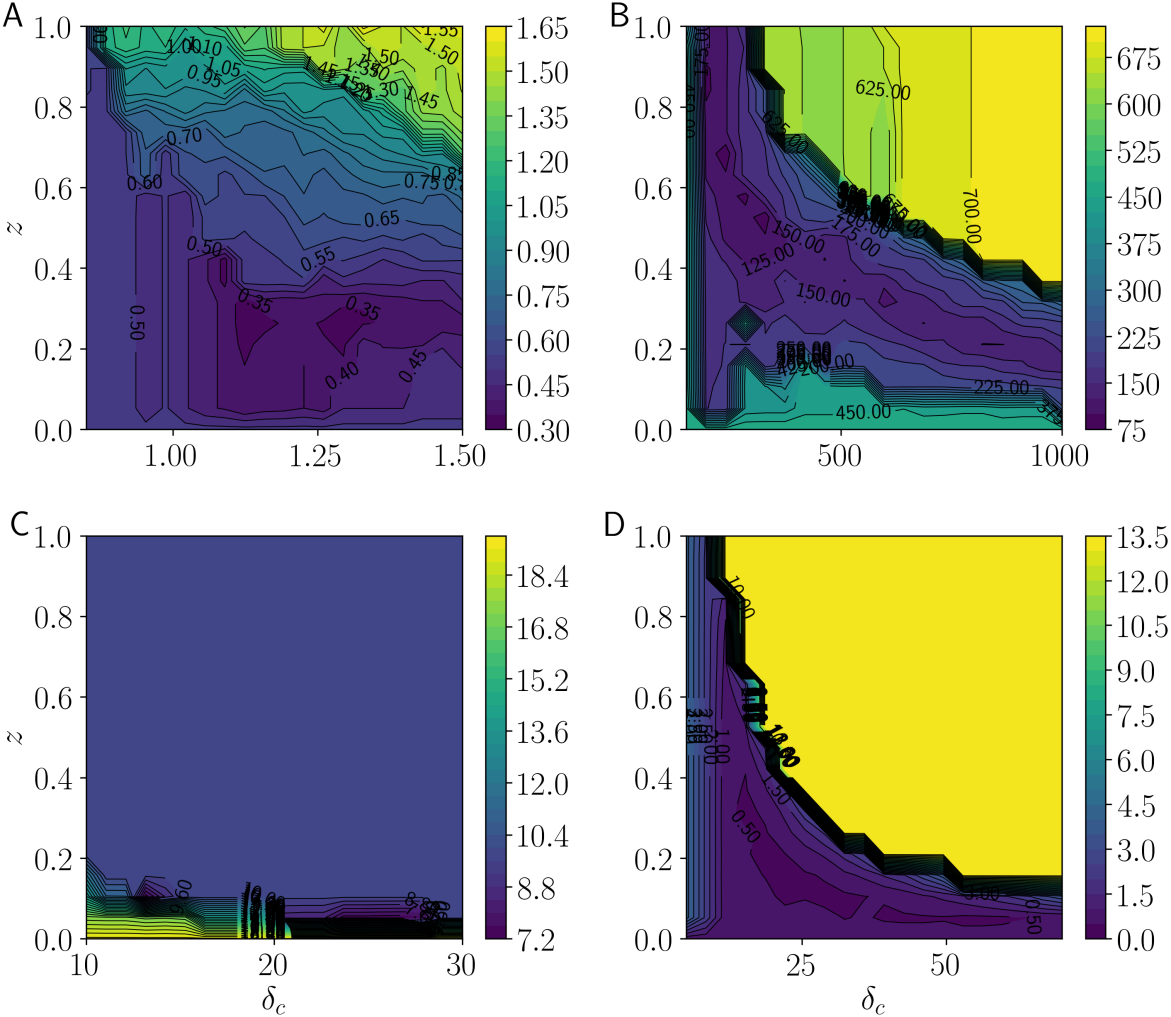
Minimum total errors between cluster centers and class centers by LiFTCM(*α* = 1 − *z*, *β* = *zδ*_*c*_, *z* ∈ [0, 1], $\overline{w}=1$) representing HCM, GNR, CNR, and their combinations: (A) Iris, (B) Wine, (C) Glass, and (D) Breast Cancer Wisconsin.

Figure 8 shows the center-error measurements as *δ*_*c*_ and *z* take 100 equally distributed values using contour lines. Figure 8A shows the results for the Iris dataset. Performance is improved at approximately *δ*_*c*_ = 1.1 and *z* = 0.3, or at approximately *δ*_*c*_ = 1.3 and *z* = 0.3. This implies that setting an appropriate noise distance and combinations of noise and possibilistic clustering yield satisfactory results. Figure 8B shows the results for the Wine dataset. Performance is improved at approximately *δ*_*c*_ = 300 and *z* = 0.5. When *δ*_*c*_ is increased, performance is maintained by decreasing *z*. This implies that general noise rejection performs better than cluster-wise noise rejection when the noise distance is large. Figure 8C shows the results for the Glass dataset. Performance is improved at approximately *δ*_*c*_ = 25 and *z* = 0.05. Among combinations, those closer to general noise rejection perform well. Figure 8D shows the results for the Breast Cancer Wisconsin dataset. Performance is improved at approximately *δ*_*c*_ = 20 and *z* = 0.2. As with the other datasets, combinations perform well. As in the case of the Wine dataset, states close to general noise rejection perform well when *δ*_*c*_ is large. Therefore, the representation of combinations of GNR and CNR by LiFTCM is satisfactory. When the noise distance is large, states close to GNR tend to yield satisfactory results.

## Discussion

### Cluster center calculation utilizing probabilistic memberships

RCM-type methods have the problem that even if the number of objects in the boundary area is small, they have unnaturally large impacts on the new cluster center compared to the objects in the lower area, because the cluster center is calculated by the convex combination of these areas. To cope with the problem, Peters proposed *π*PRCM by introducing the cluster center calculation based on the normalized membership of the membership to the upper area, which satisfies the probabilistic constraint (Peters, 2014; Peters, 2015). “*π*” is an acronym that stands for “Principle of Indifference,” in which the probability is assigned equally by dividing the number of possible clusters. Ubukata et al. proposed *π*GRCM (Ubukata et al., 2018) based on GRCM. The proposed LiFTCM has almost the same formulation as GRCM except that the condition *α* ≥ 1 is relaxed to *α* ≥ 0. Thus, *π*LiFTCM can be formulated in a similar manner to *π*GRCM by introducing the following normalized membership ${\tilde {u}}_{ci}$ of the membership to the upper area and the cluster center calculation based on ${\tilde {u}}_{ci}$:

(22) \begin{eqnarray*}& & {\tilde {u}}_{ci}= \frac{{\overline{u}}_{ci}}{\sum _{l=1}^{C}{\overline{u}}_{li}} ,\end{eqnarray*}

(23) \begin{eqnarray*}& & {\mathbi{b}}_{c}= \frac{\sum _{i=1}^{n}{\tilde {u}}_{ci}{\mathbi{x}}_{i}}{\sum _{i=1}^{n}{\tilde {u}}_{ci}} .\end{eqnarray*}

Here, attention should be paid to the following cases. In the case of *α* < 1, that is, in the case of GNR and CNR, since non-belonging of the object to any cluster is handled and thus the denominator ${\mathop{\sum }\nolimits }_{l=1}^{C}{\overline{u}}_{li}$ can become zero, it is necessary to set ${\tilde {u}}_{ci}=0$ for all clusters in such cases.

## Conclusions

In this study, as a unified approach for general noise rejection (GNR) and cluster-wise noise rejection (CNR) in hard *C*-means (HCM), we proposed linear function threshold-based *C*-means (LiFTCM) by relaxing generalized rough *C*-means (GRCM) clustering. We showed that the linear function threshold-based assignment in LiFTCM can represent GNR, CNR, and their combinations as well as GRCM. By the visualization of the classification boundaries, transitions among conventional methods based on LiFTCM and their characteristics were clarified. In the numerical experiments, the clustering performance by LiFTCM with various parameter settings was evaluated. It was demonstrated that combinations of LRCM and PRCM, or combinations of GNR and CNR by LiFTCM performed well.

We plan to investigate the relationship between the proposed method and fuzzy clustering with noise rejection. Automatic determination of parameters will also be considered.

##  Supplemental Information

10.7717/peerj-cs.238/supp-1 Supplemental Information 1 The raw data of datasets and Python codes Iris, Wine, Glass, Breast Cancer Wisconsin and Python codes (”clustering.py”: proposed method; ”GridPointDataVisualization.py”: visualization; ”evaluation_center_error.py”: numerical experiments).

## Additional Information and Declarations

### Data Availability

The following information was supplied regarding data availability: The raw datasets are available in the Supplementary File.

## References

[ref-1] Arthur D, Vassilvitskii S (2007). k-means++: the advantages of careful seeding.

[ref-2] Bezdek JC (1981). Pattern recognition with fuzzy objective function algorithms.

[ref-3] Cuesta-Albertos JA, Gordaliza A, Matrán C (1997). Trimmed *k*-means: an attempt to robustify quantizers. The Annals of Statistics.

[ref-4] Davé RN (1991). Characterization and detection of noise in clustering. Pattern Recognition Letters.

[ref-5] Davé RN, Krishnapuram R (1997). Robust clustering methods: a unified view. IEEE Transactions on Fuzzy Systems.

[ref-6] Denœux T, Kanjanatarakul O (2016). Evidential clustering: a review.

[ref-7] Denœux T, Masson M (2003). Clustering of proximity data using belief functions. Intelligent systems for information processing.

[ref-8] Denœux T, Masson M-H (2004). EVCLUS: evidential clustering of proximity data. IEEE Transactions on Systems, Man, and Cybernetics, Part B (Cybernetics).

[ref-9] Dubes RC, Jain AK (1988). Algorithms for clustering data.

[ref-10] Dunn JC (1973). A fuzzy relative of the ISODATA process and its use in detecting compact well-separated clusters. Journal of Cybernetics.

[ref-11] Edgeworth FY (1887). On observations relating to several quantities. Hermathena.

[ref-12] García-Escudero LÁ, Gordaliza A (1999). Robustness properties of k means and trimmed k means. Journal of the American Statistical Association.

[ref-13] Gauss CF (1809). Theoria Motus Corporum Coelestium in Sectionibus Conicis Solem Ambientium.

[ref-14] Hampel FR (1975). Beyond location parameters: Robust concepts and methods. Bulletin of the International Statistical Institute.

[ref-15] Huber PJ (1964). Robust estimation of a location parameter. The Annals of Mathematical Statistics.

[ref-16] Huber PJ (1981). Robust statistics.

[ref-17] Katsavounidis I, Kuo C-CJ, Zhang Z (1994). A new initialization technique for generalized Lloyd iteration. IEEE Signal Processing Letters.

[ref-18] Kaufmann L, Rousseeuw PJ, Dodge Y (1987). Clustering by means of medoids. Statistical data analysis based on the L1—norm and related methods.

[ref-19] Krishnapuram R, Keller JM (1993). A possibilistic approach to clustering. IEEE Transactions on Fuzzy Systems.

[ref-20] Krishnapuram R, Keller JM (1996). The possibilistic c-means algorithm: insights and recommendations. IEEE Transactions on Fuzzy Systems.

[ref-21] Legendre A-M (1805). Nouvelles méthodes pour la détermination des orbites des cométes.

[ref-22] Lingras P, West C (2004). Interval set clustering of web users with rough k-means. Journal of Intelligent Information Systems.

[ref-23] MacQueen J (1967). Some methods for classification and analysis of multivariate observations.

[ref-24] Maji P, Pal SK (2007a). RFCM: a hybrid clustering algorithm using rough and fuzzy sets. Fundamenta Informaticae.

[ref-25] Maji P, Pal SK (2007b). Rough set based generalized fuzzy *C*-means algorithm and quantitative indices. IEEE Transactions on Systems, Man, and Cybernetics, Part B (Cybernetics).

[ref-26] Masson M-H, Denœux T (2008). ECM: an evidential version of the fuzzy c-means algorithm. Pattern Recognition.

[ref-27] Pal NR, Pal K, Bezdek JC (1997). A mixed c-means clustering model.

[ref-28] Pal NR, Pal K, Keller J. M, Bezdek JC (2005). A possibilistic fuzzy c-means clustering algorithm. IEEE Transactions on Fuzzy Systems.

[ref-29] Pawlak Z (1982). Rough sets. International Journal of Computer & Information Sciences.

[ref-30] Pawlak Z (1991). Rough sets: theoretical aspects of reasoning about data.

[ref-31] Peters G (2006). Some refinements of rough k-means clustering. Pattern Recognition.

[ref-32] Peters G (2014). Rough clustering utilizing the principle of indifference. Information Sciences.

[ref-33] Peters G (2015). Is there any need for rough clustering?. Pattern Recognition Letters.

[ref-34] Peters G, Crespo F, Lingras P, Weber R (2013). Soft clustering–fuzzy and rough approaches and their extensions and derivatives. International Journal of Approximate Reasoning.

[ref-35] Rousseeuw PJ (1984). Least median of squares regression. Journal of the American Statistical Association.

[ref-36] Rousseeuw PJ, Leroy A (1987). Robust regression and outlier detection.

[ref-37] Ubukata S, Kato H, Notsu A, Honda K (2018). Rough set-based clustering utilizing probabilistic memberships. Journal of Advanced Computational Intelligence and Intelligent Informatics.

[ref-38] Ubukata S, Notsu A, Honda K (2017). General formulation of rough c-means clustering. International Journal of Computer Science and Network Security.

